# Supplementary material for: Effect of CB1 Receptor Deficiency on Mitochondrial Quality Control Pathways in Gastrocnemius Muscle
Source: Biology (Basel). 2024 Feb 11;13(2):116. doi: 10.3390/biology13020116 (PMC10886598; doi:10.3390/biology13020116)
Supplement: Supplementary file 1 [file biology-13-00116-s001.zip › biology-2817832-supplementary.pdf]

Supplementary material: details of western blot membranes

Figure S1

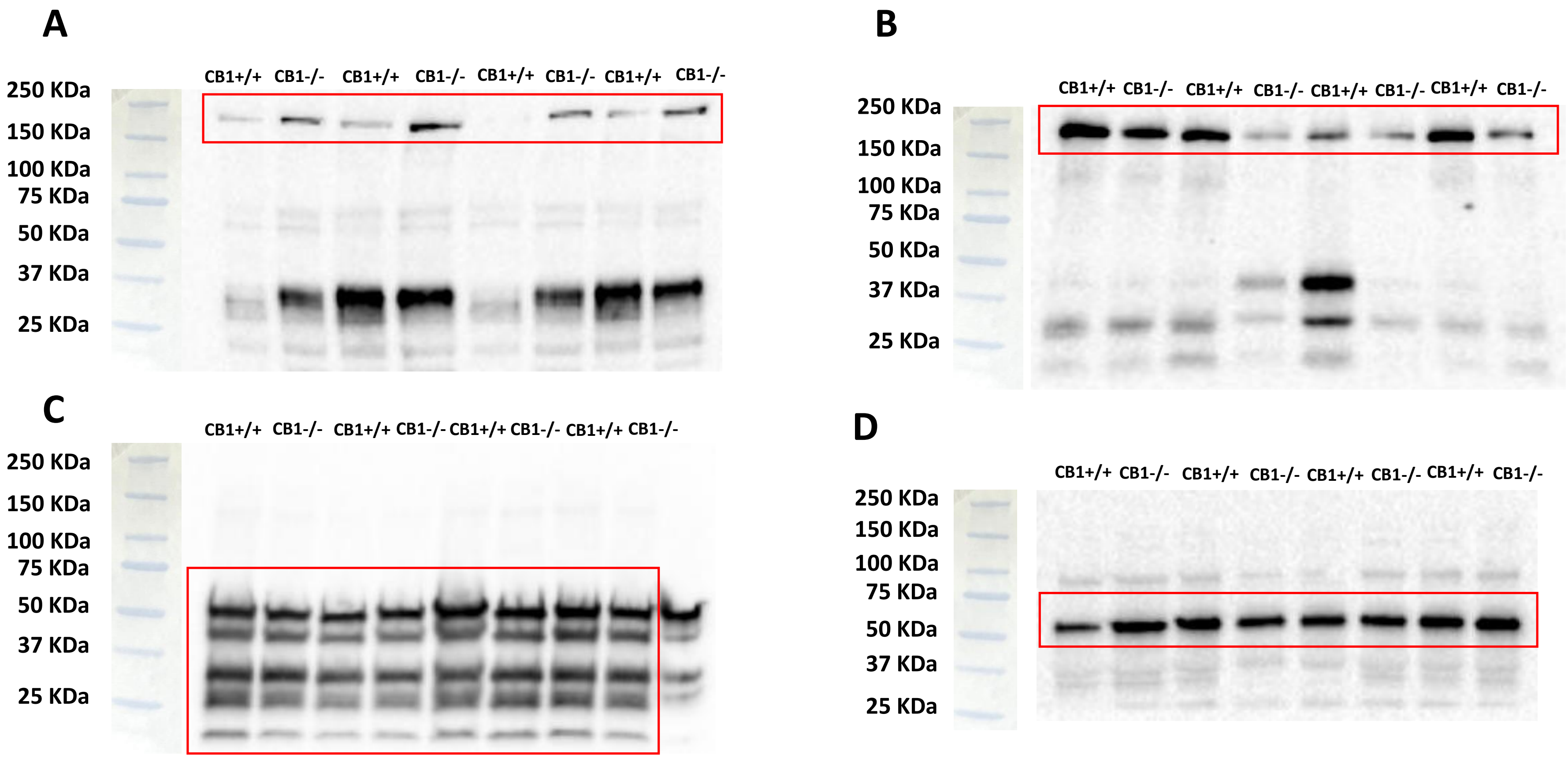

Figure S1: (A) Western blot membrane of MHCIb (~ 223 Kda) protein detected with anti-Myosin skeletal slow (NOQ7.5.4D) (GTX 11083; 1:1000; GeneTex) antibody. Gel-separated proteins were transferred to nitrocellulose membrane (0.2 µm pore size; Thermo Fisher Scientific). Membranes, incubated with a hoseradish peroxidase-conjugated secondary antibody (ab97023; 1:4000; abcam). Molecular weight (10 to 250 KDa): Precision Plus Protein™ All Blue Prestained Protein Standards (1610373; Biorad); **(B)** Western blot membrane of MHCIIb (~ 200 Kda) protein detected with anti Skeletal myosin (FAST) (M4276; 1:1000; Sigma Aldrich) antibody. Gel-separated proteins were transferred to nitrocellulose membrane (0.2 µm pore size; Thermo Fisher Scienfic). Membranes, incubated with a hoseradish peroxidase-conjugated secondary antibody (ab97023; 1:4000; abcam). Molecular weight (10 to 250 KDa): Precision Plus Protein™ All Blue Prestained Protein Standards (1610373; Biorad); **(C)** Western blot membrane of Total Oxphos (15 to 50 Kda) protein detected with Anti Total Oxphos (ab110413; 3 µg/µl; abcam) antibody. Gel-separated proteins were transferred to nitrocellulose membrane (0.2 µm pore size; Thermo Fisher Scienfic). Membranes, incubated with a hoseradish peroxidase-conjugated secondary antibody (ab97023; 1:4000; abcam). Molecular weight (10 to 250 KDa): Precision Plus Protein™ All Blue Prestained Protein Standards (1610373; Biorad); **(D)** Western blot membrane of Catalase (~ 60 Kda) protein detected with anti Catalase (C0979; 0,375 µg/ml; Sigma Aldrich) antibody. Gel-separated proteins were transferred to nitrocellulose membrane (0.2 µm pore size; Thermo Fisher Scientific). Membranes, incubated with a hoseradish peroxidase-conjugated secondary antibody (ab97023; 1:4000; abcam). Molecular weight (10 to 250 KDa): Precision Plus Protein™ All Blue Prestained Protein Standards (1610373; Biorad);

Figure S2

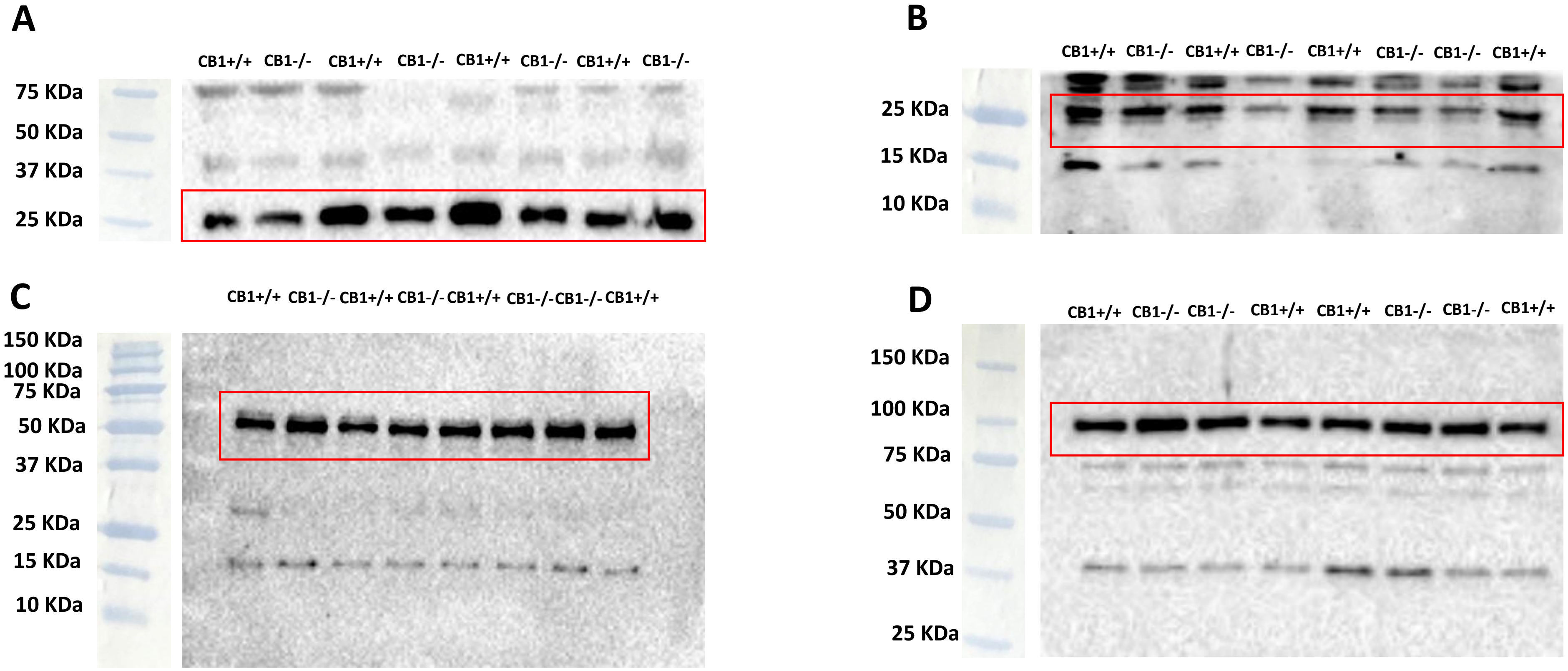

Figure S2: (A) Western blot membrane of SOD2 (~ 25 Kda) protein detected with anti- SOD2 (ab68155; 1:1000; abcam) antibody. Gel-separated proteins were transferred to nitrocellulose membrane (0.2 µm pore size; Thermo Fisher Scientific). Membranes, incubated with a hoseradish peroxidase-conjugated secondary antibody (ab97051; 1:4000; abcam). Molecular weight (10 to 250 KDa): Precision Plus Protein™ All Blue Prestained Protein Standards (1610373; Biorad); **(B)** Western blot membrane of GPX1 (~ 22 Kda) protein detected with anti- GPX1 (GTX03346; 1:1000; GeneTex) antibody. Gel-separated proteins were transferred to nitrocellulose membrane (0.2 µm pore size; Thermo Fisher Scientific). Membranes, incubated with a hoseradish peroxidase-conjugated secondary antibody (ab97051; 1:4000; abcam). Molecular weight (10 to 250 KDa): Precision Plus Protein™ All Blue Prestained Protein Standards (1610373; Biorad); **(C)** Western blot membrane of NRF1 (~ 57 Kda) protein detected with anti- NRF1 (ab34682; 1:1000; abcam) antibody. Gel-separated proteins were transferred to nitrocellulose membrane (0.2 µm pore size; Thermo Fisher Scientific). Membranes, incubated with a hoseradish peroxidase-conjugated secondary antibody (ab97051; 1:20000; abcam). Molecular weight (10 to 250 KDa): Precision Plus Protein™ All Blue Prestained Protein Standards (1610373; Biorad); **(D)** Western blot membrane of PGC1α (~ 100 Kda) protein detected with anti- PGC1α (AB3242; 1:1000; Millipore) antibody. Gel-separated proteins were transferred to nitrocellulose membrane (0.2 µm pore size; Thermo Fisher Scientific). Membranes, incubated with a hoseradish peroxidase-conjugated secondary antibody (ab97051; 1:1000; abcam). Molecular weight (10 to 250 KDa): Precision Plus Protein™ All Blue Prestained Protein Standards (1610373; Biorad);

Figure S3

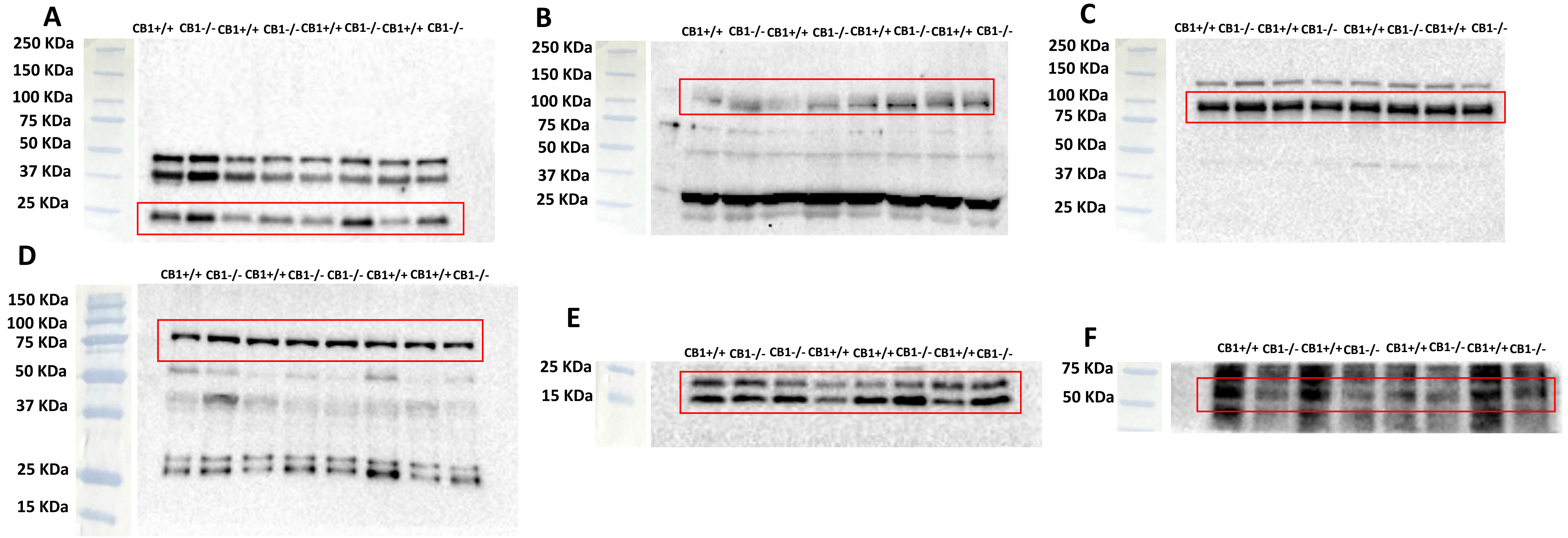

Figure S3: (A) Western blot membrane of TFAM (~ 25 Kda) protein detected with anti- mtTFA (ab131607; 1:1000; abcam) antibody. Gel-separated proteins were transferred to nitrocellulose membrane (0.2 µm pore size; Thermo Fisher Scientific). Membranes, incubated with a hoseradish peroxidase-conjugated secondary antibody (ab97051; 1:4000; abcam). Molecular weight (10 to 250 KDa): Precision Plus Protein™ All Blue Prestained Protein Standards (1610373; Biorad); **(B)** Western blot membrane of OPA1 (~ 75/112 KDa) protein detected with anti-OPA1 (ab157457; 1:1000; abcam) antibody. Gel-separated proteins were transferred to nitrocellulose membrane (0.2 µm pore size; Thermo Fisher Scientific). Membranes, incubated with a hoseradish peroxidase-conjugated secondary antibody (ab97051; 1:4000; abcam). Molecular weight (10 to 250 KDa): Precision Plus Protein™ All Blue Prestained Protein Standards (1610373; Biorad); **(C)** Western blot membrane of Drp1 (~ 78/82 KDa) protein detected with anti-Drp1 (5391; 1:1000; Cell Signaling) antibody. Gel-separated proteins were transferred to nitrocellulose membrane (0.2 µm pore size; Thermo Fisher Scientific). Membranes, incubated with a hoseradish peroxidase-conjugated secondary antibody (ab97051; 1:4000; abcam). Molecular weight (10 to 250 KDa): Precision Plus Protein™ All Blue Prestained Protein Standards (1610373; Biorad); **(D)** Western blot membrane of Mfn2 (~ 86 KDa) protein detected with anti-Mitofusin 2 (ab56889; 1:1000; abcam) antibody. Gel-separated proteins were transferred to nitrocellulose membrane (0.2 µm pore size; Thermo Fisher Scientific). Membranes, incubated with a hoseradish peroxidase-conjugated secondary antibody (ab97023; 1:4000; abcam). Molecular weight (10 to 250 KDa): Precision Plus Protein™ All Blue Prestained Protein Standards (1610373; Biorad); **(E)** Western blot membrane of LC3B (~ 15 KDa) protein detected with anti-MAP LC3β (sc-376404; 1:1000; Santa Cruz Biotechnology) antibody. Gel-separated proteins were transferred to nitrocellulose membrane (0.2 µm pore size; Thermo Fisher Scientific). Membranes, incubated with a hoseradish peroxidase-conjugated secondary antibody (ab97023; 1:4000; abcam). Molecular weight (10 to 250 KDa): Precision Plus Protein™ All Blue Prestained Protein Standards (1610373; Biorad); **(F)** Western blot membrane of p62 (~ 62 KDa) protein detected with anti-SQSTM1/p62 (5114; 1:1000; Cell Signaling) antibody. Gel-separated proteins were transferred to nitrocellulose membrane (0.2 µm pore size; Thermo Fisher Scientific). Membranes, incubated with a hoseradish peroxidase-conjugated secondary antibody (ab97051; 1:4000; abcam). Molecular weight (10 to 250 KDa): Precision Plus Protein™ All Blue Prestained Protein Standards (1610373; Biorad); (F)

Figure S4

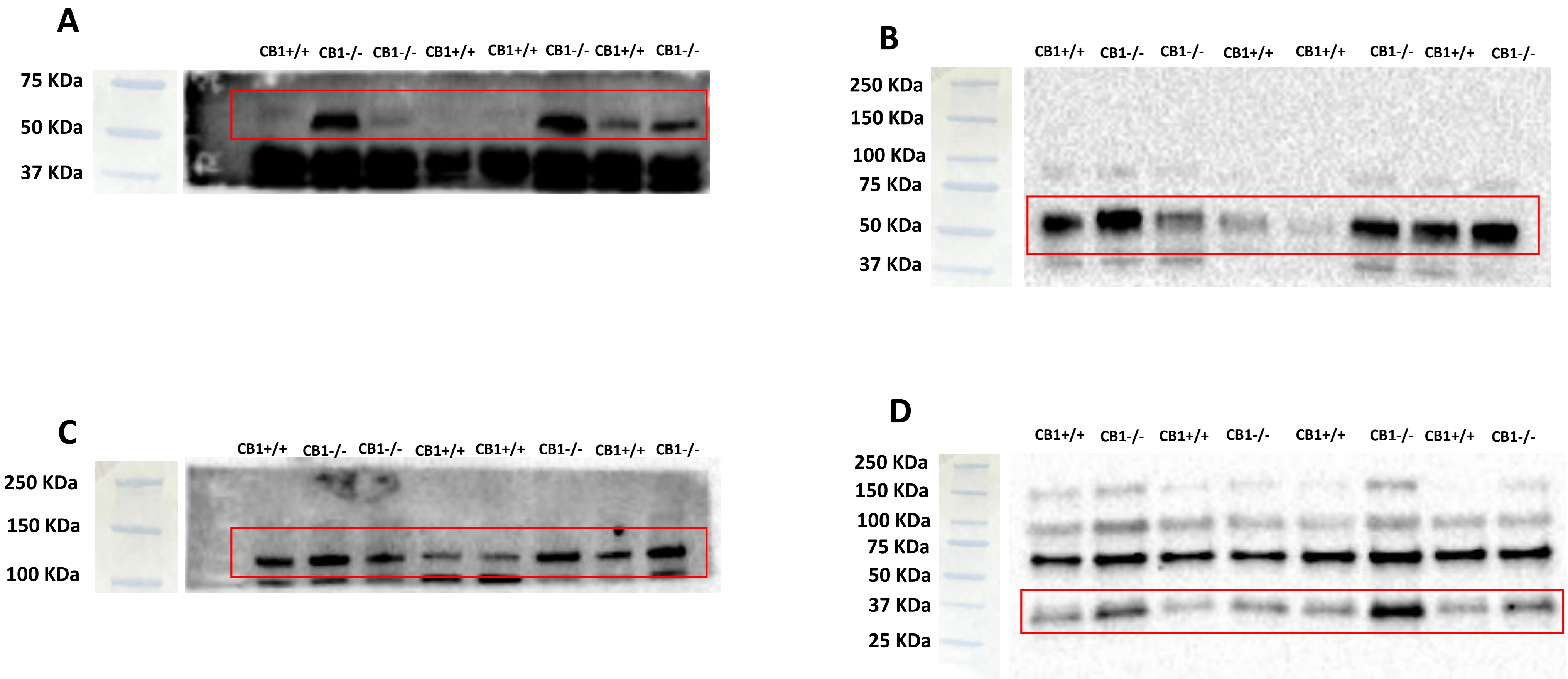

Figure S4: (A) Western blot membrane of Parkin (~ 50 Kda) protein detected with anti-Parkin (4211; 1:1000; Cell Signaling) antibody. Gel-separated proteins were transferred to nitrocellulose membrane (0.2 µm pore size; Thermo Fisher Scientific). Membranes, incubated with a hoseradish peroxidase-conjugated secondary antibody (ab97023; 1:4000; abcam). Molecular weight (10 to 250 KDa): Precision Plus Protein™ All Blue Prestained Protein Standards (1610373; Biorad); **(B)** Western blot membrane of Pink1 (~ 50 KDa) protein detected with anti-PINK1 (ab186303; 1:1000; abcam) antibody. Gel-separated proteins were transferred to nitrocellulose membrane (0.2 µm pore size; Thermo Fisher Scientific). Membranes, incubated with a hoseradish peroxidase-conjugated secondary antibody (ab97023; 1:4000; abcam). Molecular weight (10 to 250 KDa): Precision Plus Protein™ All Blue Prestained Protein Standards (1610373; Biorad); **(C)** Western blot membrane of Ambra1 (~ 135/150 KDa) protein detected with anti-Ambra1 (24907; 1:1000; Cell Signaling) antibody. Gel-separated proteins were transferred to nitrocellulose membrane (0.2 µm pore size; Thermo Fisher Scientific). Membranes, incubated with a hoseradish peroxidase-conjugated secondary antibody (ab97051; 1:4000; abcam). Molecular weight (10 to 250 KDa): Precision Plus Protein™ All Blue Prestained Protein Standards (1610373; Biorad); **(D)** Western blot membrane of APE1 (~ 135/150 KDa) protein detected with anti-APE (13B8E5C2; 1:1000; Novus Biologicals) antibody. Gel-separated proteins were transferred to nitrocellulose membrane (0.2 µm pore size; Thermo Fisher Scientific). Membranes, incubated with a hoseradish peroxidase-conjugated secondary antibody (ab97023; 1:4000; abcam). Molecular weight (10 to 250 KDa): Precision Plus Protein™ All Blue Prestained Protein Standards (1610373; Biorad);

Figure S5

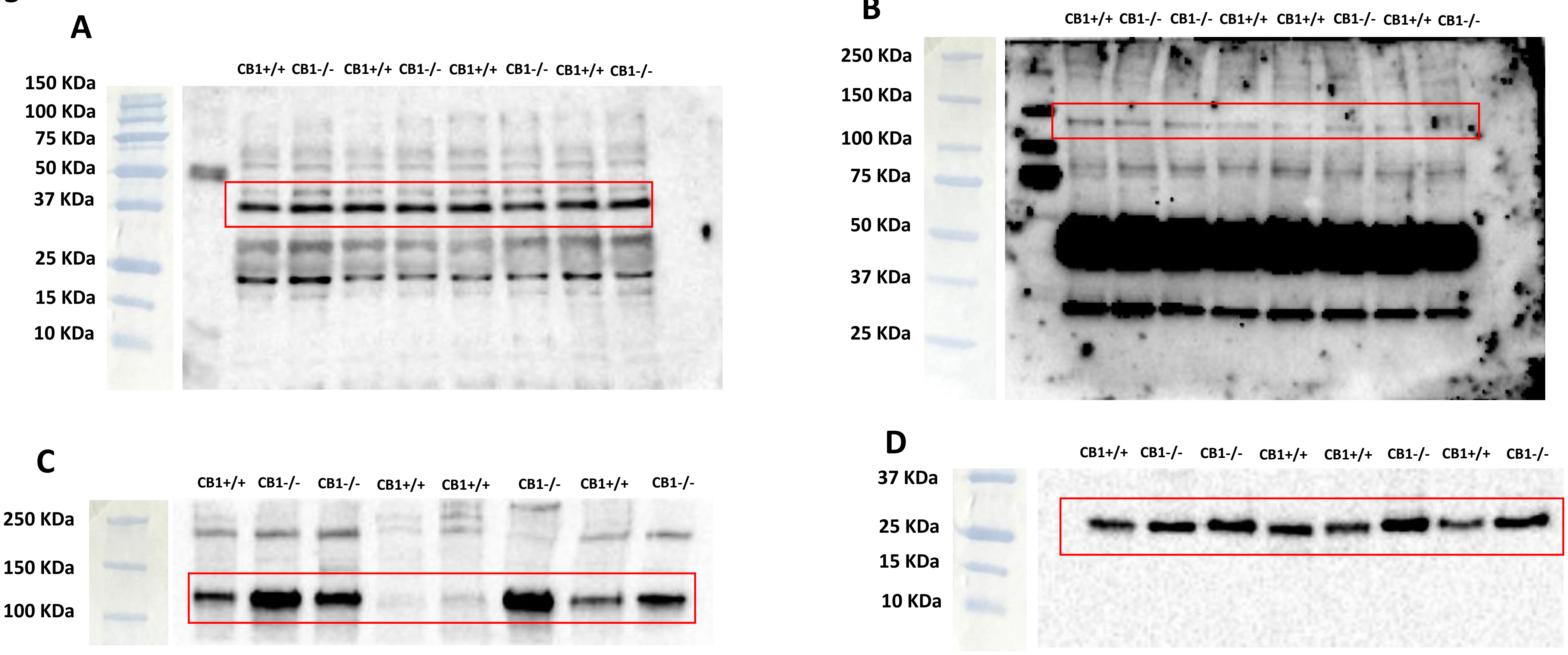

Figure S5: (A) Western blot membrane of OGG1 (~ 39 KDa) protein detected with anti-OGG1 (NB100-106; 1:1000; Novus Biologicals) antibody. Gel-separated proteins were transferred to nitrocellulose membrane (0.2 µm pore size; Thermo Fisher Scientific). Membranes, incubated with a hoseradish peroxidase-conjugated secondary antibody (ab97051; 1:4000; abcam). Molecular weight (10 to 250 KDa): Precision Plus Protein™ All Blue Prestained Protein Standards (1610373; Biorad); **(B)** Western blot membrane of POL-γ (~ 140 KDa) protein detected with anti-DNA Pol-γ (sc-5931; 1:1000; Santa Cruz Biotechnology) antibody. Gel-separated proteins were transferred to nitrocellulose membrane (0.2 µm pore size; Thermo Fisher Scientific). Membranes, incubated with a hoseradish peroxidase-conjugated secondary antibody. Molecular weight (10 to 250 KDa): Precision Plus Protein™ All Blue Prestained Protein Standards (1610373; Biorad); **(C)** Western blot membrane of LONP1 (~ 106 KDa) protein detected with anti-LONP1 (A4293; 1:1000; ABclonal) antibody. Gel-separated proteins were transferred to nitrocellulose membrane (0.2 µm pore size; Thermo Fisher Scientific). Membranes, incubated with a hoseradish peroxidase-conjugated secondary antibody (ab97051; 1:4000; abcam). Molecular weight (10 to 250 KDa): Precision Plus Protein™ All Blue Prestained Protein Standards (1610373; Biorad); **(D)** Western blot membrane of CLPP (~ 26 KDa) protein detected with anti-CLPP (A3214; 1:1000; ABclonal) antibody. Gel-separated proteins were transferred to nitrocellulose membrane (0.2 µm pore size; Thermo Fisher Scientific). Membranes, incubated with a hoseradish peroxidase-conjugated secondary antibody (ab97051; 1:4000; abcam). Molecular weight (10 to 250 KDa): Precision Plus Protein™ All Blue Prestained Protein Standards (1610373; Biorad);

Figure S6

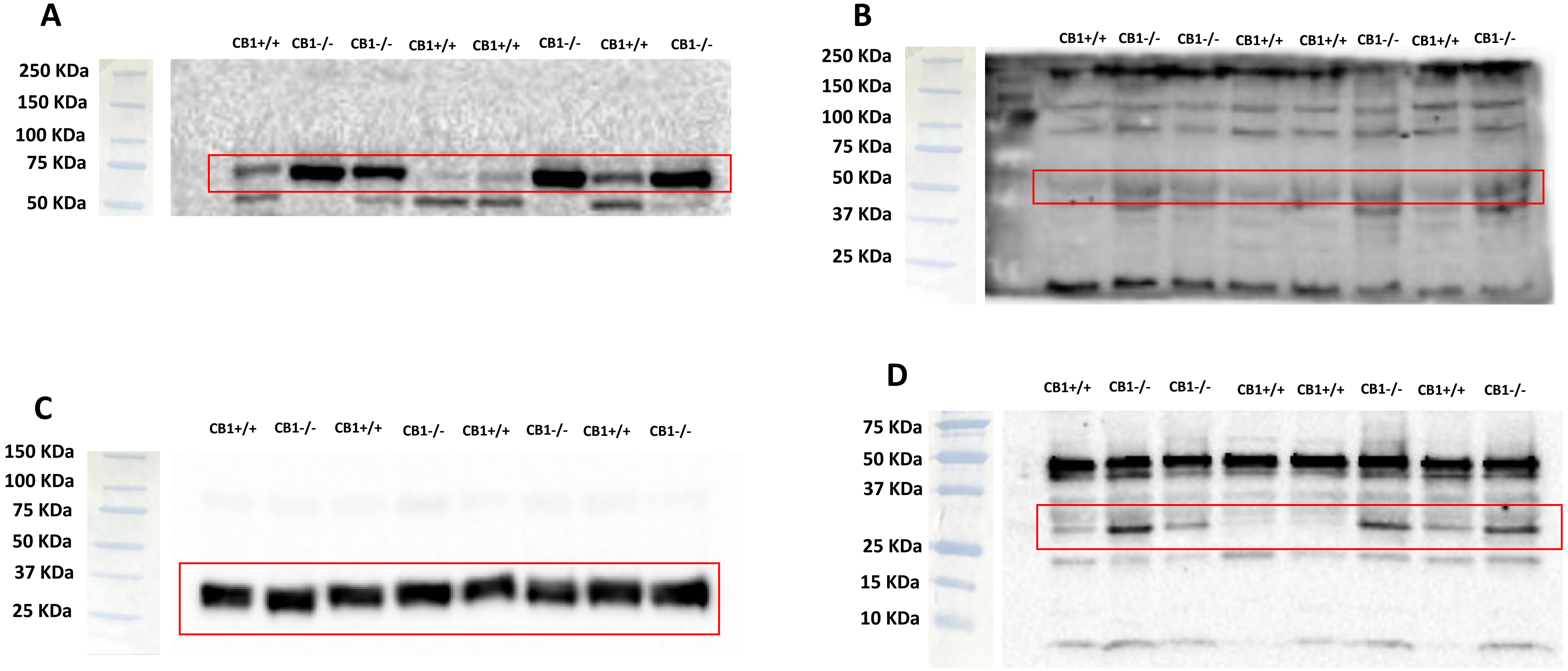

Figure S6: (A) Western blot membrane of TRAP1 (~ 80 KDa) protein detected with anti-TRAP1 (A2748; 1:1000; ABclonal) antibody. Gel-separated proteins were transferred to nitrocellulose membrane (0.2 µm pore size; Thermo Fisher Scientific). Membranes, incubated with a hoseradish peroxidase-conjugated secondary antibody (ab97051; 1:4000; abcam). Molecular weight (10 to 250 KDa): Precision Plus Protein™ All Blue Prestained Protein Standards (1610373; Biorad); **(B)** Western blot membrane of ATF4 (~ 50 KDa) protein detected with anti-ATF4 (A18687; 1:1000;ABclonal) antibody. Gel-separated proteins were transferred to nitrocellulose membrane (0.2 µm pore size; Thermo Fisher Scientific). Membranes, incubated with a hoseradish peroxidase-conjugated secondary antibody (ab97051; 1:4000; abcam). Molecular weight (10 to 250 KDa): Precision Plus Protein™ All Blue Prestained Protein Standards (1610373; Biorad); **(C)** Western blot membrane of ATF5 (~ 35 KDa) protein detected with anti-ATF5 (A3563; 1:1000; ABclonal) antibody. Gel-separated proteins were transferred to nitrocellulose membrane (0.2 µm pore size; Thermo Fisher Scientific). Membranes, incubated with a hoseradish peroxidase-conjugated secondary antibody (ab97051; 1:4000; abcam). Molecular weight (10 to 250 KDa): Precision Plus Protein™ All Blue Prestained Protein Standards (1610373; Biorad); **(D)** Western blot membrane of CHOP (~ 27 KDa) protein detected with anti-DDIT3/CHOP (A0221; 1:1000; ABclonal) antibody. Gel-separated proteins were transferred to nitrocellulose membrane (0.2 µm pore size; Thermo Fisher Scientific). Membranes, incubated with a hoseradish peroxidase-conjugated secondary antibody (ab97051; 1:4000; abcam). Molecular weight (10 to 250 KDa): Precision Plus Protein™ All Blue Prestained Protein Standards (1610373; Biorad);

Figure S7

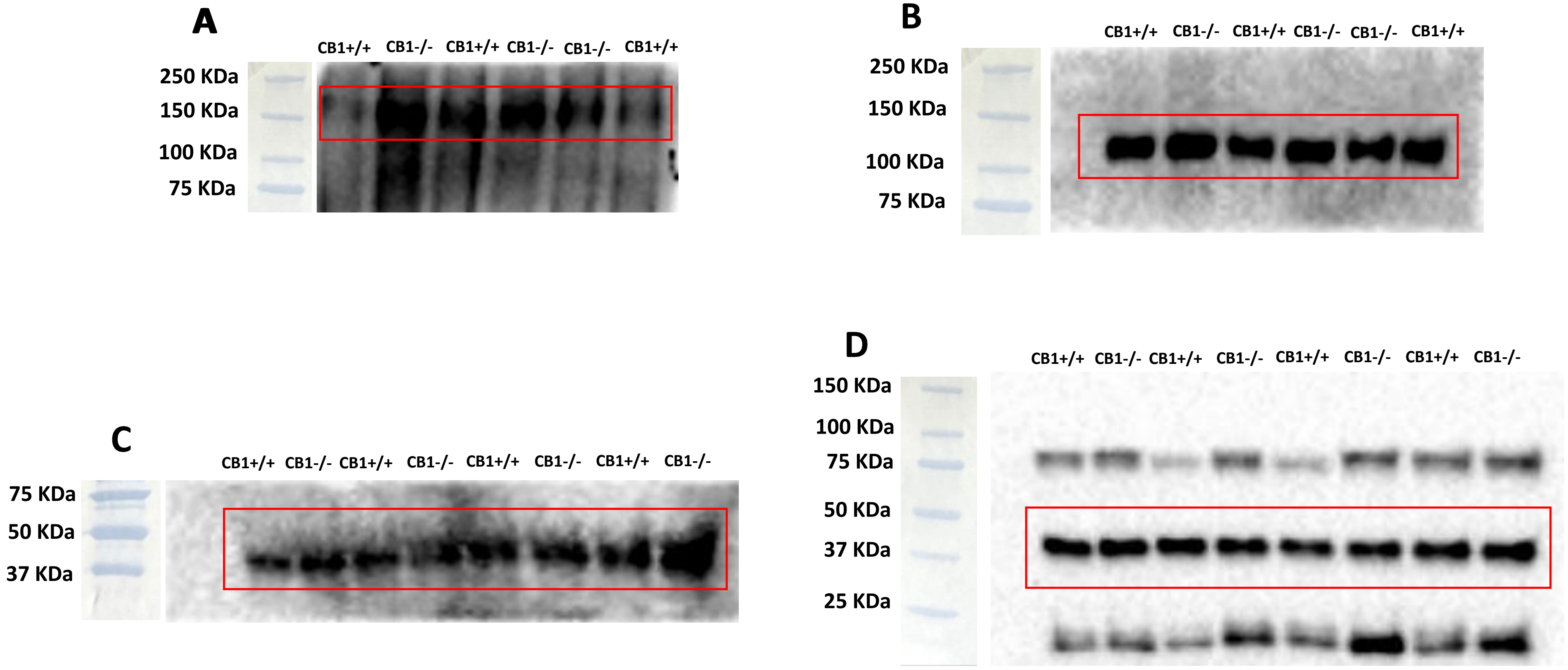

Figure S7: (A) Western blot membrane of P-PERK (Thr980) (~ 170 KDa) protein detected with anti- P-PERK (Thr980) (3179; 1:1000; Cell Signaling) antibody. Gel-separated proteins were transferred to nitrocellulose membrane (0.2 µm pore size; Thermo Fisher Scientific). Membranes, incubated with a hoseradish peroxidase-conjugated secondary antibody (ab97051; 1:4000; abcam). Molecular weight (10 to 250 KDa): Precision Plus Protein™ All Blue Prestained Protein Standards (1610373; Biorad); **(B)** Western blot membrane of PERK (~ 140 KDa) protein detected with anti-PERK (3192; 1:1000; Cell Signaling) antibody. Gel-separated proteins were transferred to nitrocellulose membrane (0.2 µm pore size; Thermo Fisher Scientific). Membranes, incubated with a hoseradish peroxidase-conjugated secondary antibody (ab97051; 1:4000; abcam). Molecular weight (10 to 250 KDa): Precision Plus Protein™ All Blue Prestained Protein Standards (1610373; Biorad); **(C)** Western blot membrane of P-eiF2α (Ser51) (~ 38 KDa) protein detected with anti-P-eiF2α (Ser51) (3398; 1:1000; Cell Signaling) antibody. Gel-separated proteins were transferred to nitrocellulose membrane (0.2 µm pore size; Thermo Fisher Scientific). Membranes, incubated with a hoseradish peroxidase-conjugated secondary antibody (ab97051; 1:4000; abcam). Molecular weight (10 to 250 KDa): Precision Plus Protein™ All Blue Prestained Protein Standards (1610373; Biorad); **(D)** Western blot membrane of eiF2α (~ 38 KDa) protein detected with anti-eiF2α (2103; 1:1000; Cell Signaling) antibody. Gel-separated proteins were transferred to nitrocellulose membrane (0.2 µm pore size; Thermo Fisher Scientific). Membranes, incubated with a hoseradish peroxidase-conjugated secondary antibody (ab97023; 1:4000; abcam). Molecular weight (10 to 250 KDa): Precision Plus Protein™ All Blue Prestained Protein Standards (1610373; Biorad);

Figure S8

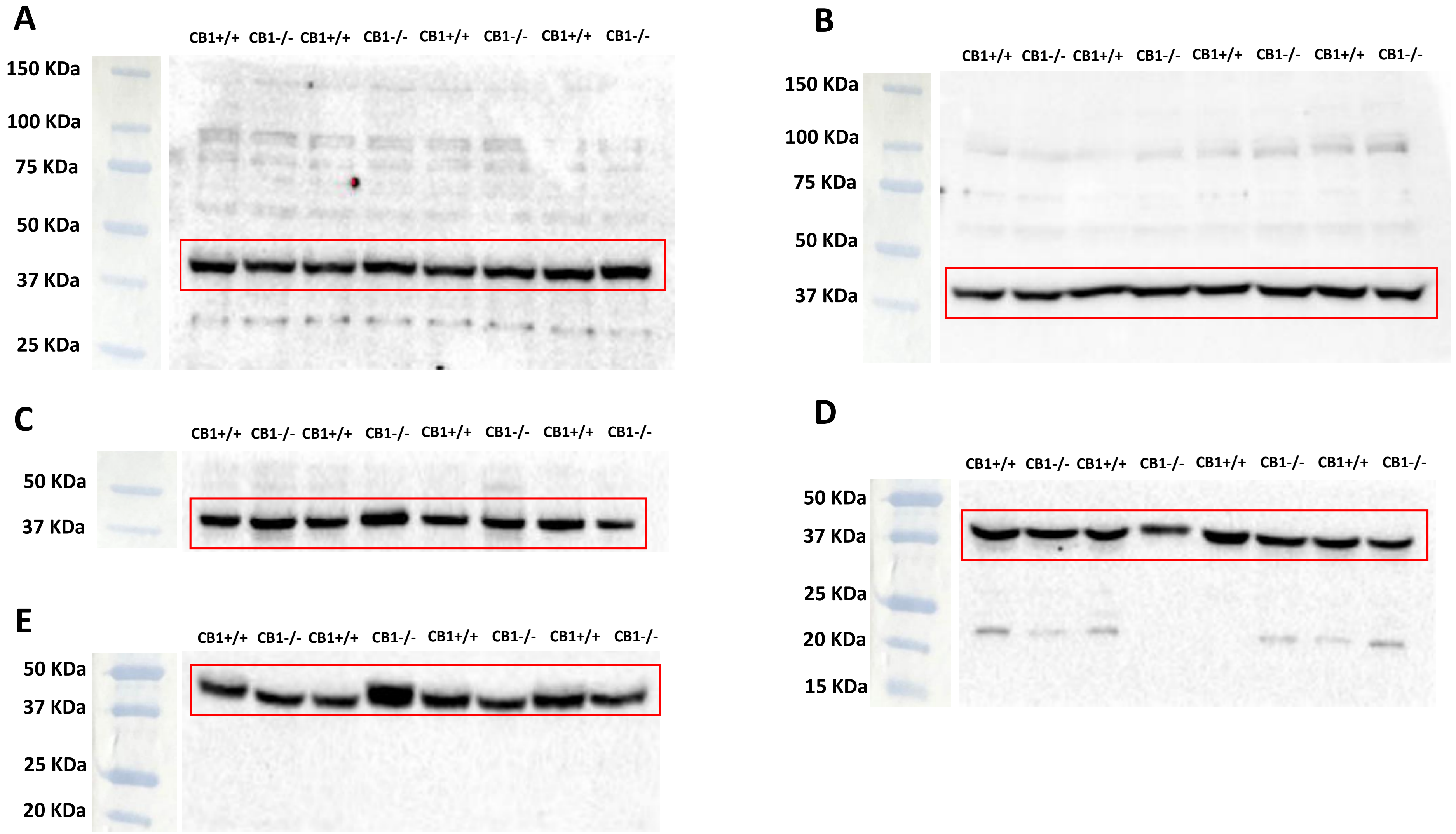

Figure S8: (A), (B), (C), (D) and **(E)** Western blot membrane of B-ACTIN (~ 43 KDa) protein detected with anti- B-ACTIN (bs-0061R; 1:1000; BIOSS ANTIBODIES) antibody. Gel-separated proteins were transferred to nitrocellulose membrane (0.2 µm pore size; Thermo Fisher Scientific). Membranes, incubated with a hoseradish peroxidase-conjugated secondary antibody (ab97051; 1:4000; abcam). Molecular weight (10 to 250 KDa): Precision Plus Protein™ All Blue Prestained Protein Standards (1610373; Biorad);

Figure S9

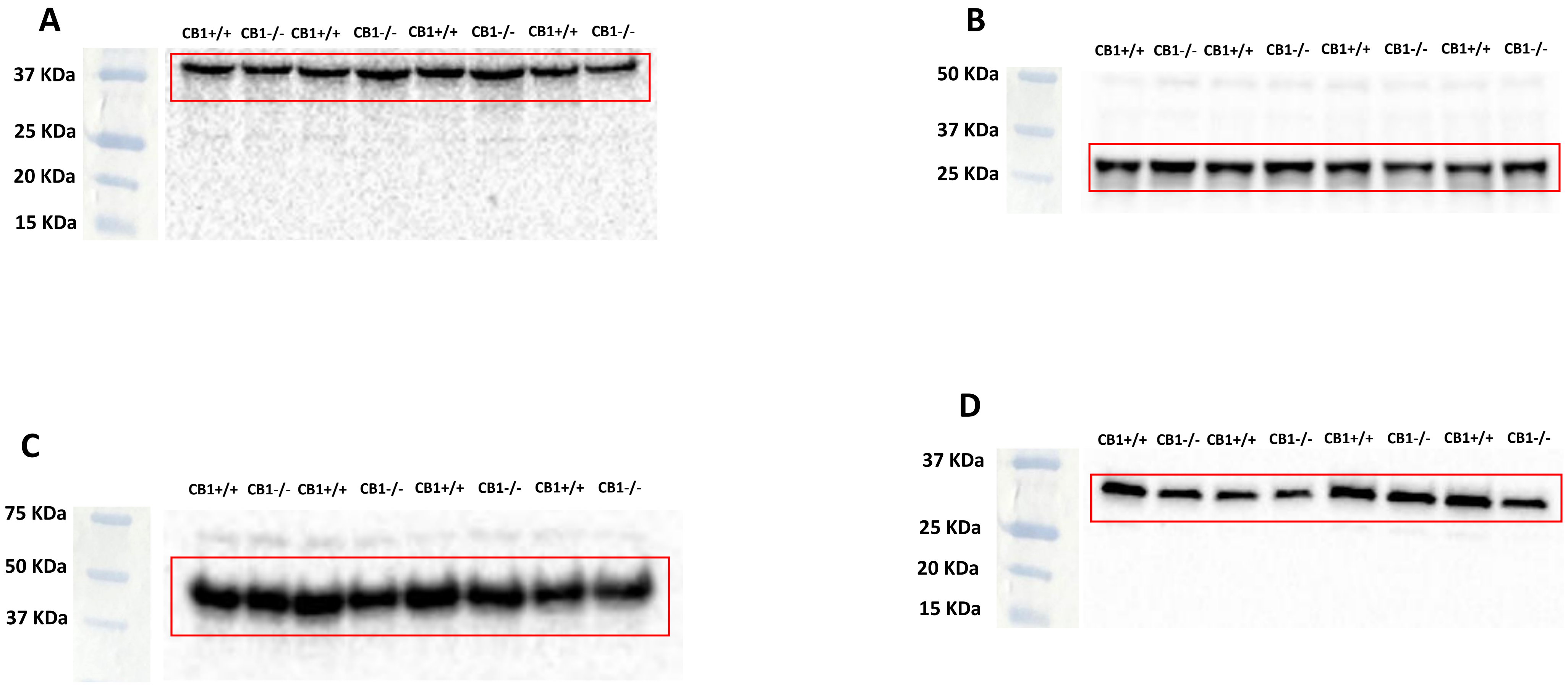

Figure S9: (A), (B) and **(C)** Western blot membrane of B-ACTIN (~ 43 KDa) protein detected with anti- B-ACTIN (bs-0061R; 1:1000; BIOSS ANTIBODIES) antibody. Gel-separated proteins were transferred to nitrocellulose membrane (0.2 µm pore size; Thermo Fisher Scientific). Membranes, incubated with a hoseradish peroxidase-conjugated secondary antibody (ab97051; 1:4000; abcam). Molecular weight (10 to 250 KDa): Precision Plus Protein™ All Blue Prestained Protein Standards (1610373; Biorad); **(D)** Western blot membrane of VDAC1 (~ 31 KDa) protein detected with anti- VDAC1 (GTX114187; 1:1000; GeneTex) antibody. Gel-separated proteins were transferred to nitrocellulose membrane (0.2 µm pore size; Thermo Fisher Scientific). Membranes, incubated with a hoseradish peroxidase-conjugated secondary antibody (ab97051; 1:4000; abcam). Molecular weight (10 to 250 KDa): Precision Plus Protein™ All Blue Prestained Protein Standards (1610373; Biorad);
